# MicroRNA Regulation of Host Immune Responses following Fungal Exposure

**DOI:** 10.3389/fimmu.2018.00170

**Published:** 2018-02-07

**Authors:** Tara L. Croston, Angela R. Lemons, Donald H. Beezhold, Brett J. Green

**Affiliations:** ^1^Allergy and Clinical Immunology Branch, Health Effects Laboratory Division, National Institute for Occupational Safety and Health, Centers for Disease Control and Prevention, Morgantown, WV, United States; ^2^Health Effects Laboratory Division, National Institute for Occupational Safety and Health, Centers for Disease Control and Prevention, Morgantown, WV, United States

**Keywords:** fungal exposure, microRNA, fungi, immune response, inflammatory response

## Abstract

Fungal bioaerosols are ubiquitous in the environment and human exposure can result in a variety of health effects ranging from systemic, subcutaneous, and cutaneous infections to respiratory morbidity including allergy, asthma, and hypersensitivity pneumonitis. Recent research has focused on the role of microRNAs (miRNAs) following fungal exposure and is overlooked, yet important, group of regulators capable of influencing fungal immune responses through a variety of cellular mechanisms. These small non-coding ribose nucleic acids function to regulate gene expression at the post-transcriptional level and have been shown to participate in multiple disease pathways including cancer, heart disease, apoptosis, as well as immune responses to microbial hazards and occupational allergens. Recent animal model studies have characterized miRNAs following the exposure to inflammatory stimuli. Studies focused on microbial exposure, including bacterial infections, as well as exposure to different allergens have shown miRNAs, such as miR-21, miR-146, miR-132, miR-155, and the let-7 family members, to be involved in immune and inflammatory responses. Interestingly, the few studies have assessed that the miRNA profiles following fungal exposure have identified the same critical miRNAs that have been characterized in other inflammatory-mediated and allergy-induced experimental models. Review of available *in vitro*, animal and human studies of exposures to *Aspergillus fumigatus, Candida albicans, Cryptococcus neoformans, Paracoccidioides brasiliensis*, and *Stachybotrys chartarum* identified several miRNAs that were shared between responses to these species including miR-125 a/b (macrophage polarization/activation), miR-132 [toll-like receptor (TLR)2-mediated signaling], miR-146a (TLR mediated signaling, alternative macrophage activation), and miR-29a/b (natural killer cell function, C-leptin signaling, inhibition of Th1 immune response). Although these datasets provide preliminary insight into the role of miRNAs in fungal exposed models, interpretation of miRNA datasets can be challenging for researchers. To assist in navigating this rapidly evolving field, the aim of this review is to describe miRNAs in the framework of host recognition mechanisms and provide initial insight into the regulatory pathways in response to fungal exposure.

## Introduction

Fungi are ubiquitous eukaryotic microorganisms that can be prevalent in indoor, outdoor, and occupational environments. A small portion of 1.5 million fungal species estimated to exist (1) are primary or opportunistic pathogens, whereas the vast majority is ubiquitous saprophytes that obtain nutrients from organic matter. Fungi are composed of membrane bound organelles that are encased by a rigid cell wall but do not contain chlorophyll. The cell wall is composed of ergosterol, chitin, glucans, such as (1 → 3)-β-d-glucan, and mannose proteins (2). Fungal lifeforms broadly vary from unicellular yeasts to multicellular filamentous hyphae that include the production of mitotic or meiotically produced spores. In some cases, fungi are dimorphic and share both lifecycles. Upon disturbance, fungal spores can be aerosolized and in some occupational environments the airborne concentrations may exceed 1 × 10^5^ spores/m^3^ (3).

Personal exposure to fungal species has been associated with a broad variety of adverse health effects that range from pulmonary, sinus, and subcutaneous infections to respiratory morbidities that may include hypersensitivity pneumonitis, allergy, and asthma (3). Each of these health effects is dependent on the host’s immune responsiveness and fungal species exposed (4). In specific geographical regions, dimorphic fungi that cause endemic mycoses exist as either a filamentous fungus in the environment or as a pathogenic yeast in the host. In the environment, the filamentous hyphae grow in soil at ambient temperatures and produce infectious spores (5, 6). Soil disturbance can aerosolize spores that can be inhaled by a mammalian host. In a process that is thermo-regulated, the spores can germinate into a pathogenic yeast phase that helps these fungi avoid the hosts’ immune responses. For example, *Blastomyces dermatitidis* can proliferate on the respiratory mucosa, *Histoplasma capsulatum* modulates the monocyte phagolysosome compartment, and *Coccidioides immitis* develops a large spherule containing endospores that is resistant to phagocytosis. These dimorphic fungal species as well as others, including *Paracoccidioides brasiliensis* (paracoccidioidomycosis) and *Talaromyces* (*Penicillium*) *marneffei* (talaromycosis), affect the lungs, although the latter can also affect the liver and mouth.

By contrast, opportunistic fungal pathogens consist of fungi that are environmentally ubiquitous and affect those who are immunocompromised, especially patients who have received a transplant or undergoing chemotherapy or corticosteroid therapy. Examples of fungi that are commonly implicated in opportunistic infections include, *Candida albicans* (candidiasis), *Pneumocystis jirovecii* (*Pneumocystis* pneumonia), *Cryptococcus neoformans/gattii* (cryptococcosis), and *Aspergillus fumigatus* (aspergillosis). Infections can be acquired through the inhalation of conidia or yeast depending on the species and can result in systemic mycoses. With the increase in broad-spectrum antibiotic usage and other medical and therapeutic strategies, invasive opportunistic fungal infections are of particular concern in the hospital setting, as nosocomial infections may be life-threatening for critically ill individuals (7).

The World Health Organization and the Institute of Medicine have published consensus documents that report respiratory morbidities are associated with damp indoor environments (8, 9). Recent epidemiological evidence has further built on these consensus findings and shown exposure to mold in damp indoor environments to be associated with adverse respiratory health effects (10, 11). Following recent natural disasters and flooding events associated with Hurricanes Harvey, Irma, and Maria, water-infiltrated occupational, and residential buildings are environments where mold can grow and proliferate on water damaged building materials. Returning to these environments and disturbing contaminated building materials can result in the aerosolization of fungal spores (12) that can pose a significant health risk especially if the person is immunocompromised. Fungi associated with colonizing wet building materials include, *Aspergillus versicolor, Ulocladium chartarum, Chaetomium globosum*, and *Stachybotrys chartarum* that are hydrophilic and require a high water activity for growth and proliferation. Of these hydrophilic fungal contaminants, *S. chartarum* is the most widely studied and many reports have identified exposure to contribute to negative health effects (12–15).

Due to increased community concern regarding personal exposure to these pathogenic fungi and the potential result of life-threatening health outcomes, it is important to characterize the mechanisms that contribute to the host innate and adaptive immune responses. Previous research has focused on host responses in fungal exposure models by analyzing functional, histological, and immunological endpoints; however, research examining the molecular mechanisms that underlie these responses remains unclear for many clinically relevant fungal species. Although many studies have been published that have explored pulmonary immunological responses to acute and chronic fungal spores exposures, the microRNAs (miRNAs) that regulate these deficiencies have not been fully characterized. In this review, the state-of-knowledge of miRNAs characterized in various animal models, including those that have evaluated fungal exposures, will be reviewed with emphasis placed on the mechanistic insights that these studies have provided in relation to the host response following fungal exposure.

## MicroRNAs

MicroRNAs are an important group of regulators capable of influencing gene expression through different mechanisms (16–20). Consisting of short, single stranded noncoding ribose nucleic acids (RNAs), miRNAs bind to target messenger RNA (mRNA) to downregulate gene expression post-transcriptionally through RNA silencing or RNA degradation (21, 22). Depending on the complementarity of base pairing, gene expression is repressed, as observed in humans and animals, or mRNA is cleaved, as observed in plants (23–25). More recently, studies have shown that miRNA can also activate the translation of certain target mRNA (17, 18, 26). Providing insight into how altered miRNA profiles affect upstream processes can be methodologically challenging. For example, a single miRNA can regulate from one to multiple genes, whereas studies have also shown that multiple miRNAs can regulate the same gene (27–30). Several miRNAs, as well as miRNA families, have been extensively studied and have been characterized in models of cancer, heart disease, aging, apoptosis, and immune responses to inflammatory stimuli (19, 20, 31–34).

### Influence of miRNAs on IL-13-Mediated Allergic Responses

The *let-7* family is the most abundant pulmonary miRNAs and has been identified in cancer, diabetes, and aging studies (35–39). The *let-7* family members have been shown to target interleukin (IL)-13 in *in vivo* and *in vitro* models, although the regulatory *in vivo* mechanisms of *let-7* are complex (20, 40). miR-21 is another widely studied miRNA and has been shown to participate in the inflammatory response elicited by different stimuli, including doxycycline-induced allergic airway inflammation (41), as well as viral, bacterial and protozoan infections (42–44). One of the most upregulated miRNAs in human patients with allergic eosinophilic esophagitis is miR-21 (45, 46), which correlates with studies that reported miR-21 and miR-223 as regulators of eosinophilic development in an *ex vivo* model of bone-derived eosinophils (47, 48). miR-375 has also been reported to be downregulated in epithelial cells derived from patients with eosinophilic esophagitis, as well as in IL-13 stimulated epithelial cells indicating the role of miR-375 as a regulator of IL-13-mediated responses (21).

### miRNA Involvement in Allergy-Induced Asthma

In rodent models exposed to house dust mite allergen, increased miR-126, miR-106a, and miR-145 expression have been shown to contribute to allergic inflammation (49–51). Studies involving airborne pollutants, such as cigarette smoke, reported a downregulation in let-7c, let-7f, miR-34b, miR-34c, and miR-222, all of which contributed to pulmonary inflammation in rodent models (52–54). Research examining aberrant miRNA profiles in asthmatic hosts has also revealed novel miRNAs that contribute to allergic airway disease. Examination of CD4^+^ T cells isolated from the bronchoalveolar lavage fluid from asthmatic human patients revealed that miR-19a had the highest expression (55), which promoted a Th2-mediated cell response, a known response contributing to allergic asthma. In another study, miR-221 and miR-485-3p were upregulated in peripheral blood from pediatric asthmatic human patients compared with controls, suggesting that these miRNAs contribute to the development of asthma (56). In a chemical allergen model examining the murine miRNA profile following dermal exposure to toluene 2,4-diisocyanate, miR-21, miR-22, miR-27b, miR-31, miR-126, miR-155, miR-210, and miR-301a expression were increased (57). While this study identified miRNAs that are known to participate in the immune response associated with asthma (miR-21, miR-31, miR-126, and miR-155), new miRNAs were proposed as potential biomarkers for allergic sensitization to toluene 2,4-diisocyanate (miR-22, miR-27b, miR-301a, and miR-210).

### miRNA Regulation on Adaptive Immunity

MicroRNAs critically influence the development and responses of the immune system, but the contributing biological mechanisms are poorly characterized (22, 58–60). Overexpression of the miR-17-92 cluster and miR-181 enhanced B-cell proliferation, while miR-150 regulated B-cell differentiation (61–64). When overexpressed, miR-181 has been shown to decrease T-cell numbers (61), but enhance T-cell receptor signaling (65). When T cells are activated, the miRNA expression profiles are altered (66–68). T-cell activation has additionally been found to induce the miR-17~92 family members (69), as well as the gene that encodes miR-155 (70). miR-155 has also been reported to regulate antigen presentation (71) and to negatively regulate toll-like receptor (TLR) and cytokine signaling (72). The miR-17-92 cluster promotes Th1 type immune responses along with inhibiting regulatory T-cell differentiation (69). Rodriquez et al. showed that miR-155 is required for normal functioning of B and T lymphocytes as well as dendritic cells (73).

### Macrophage Development and TLR Signaling

In human macrophages, miR-155 targets and subsequently decreases IL-13Rα1, modulating the IL-13 pathway and the switching between classic and alternatively activated macrophages (74). Macrophage polarization is transcriptionally controlled by either miR-146b or miR-34a, directing an M1 macrophage polarization, whereas miR-18a/miR-34a, miR-130b, or miR-125-5p dictates an M2 macrophage phenotype (75). miR-21 has also been reported to direct macrophage polarization from an M1 phenotype toward an M2 phenotype (75). Alveolar macrophages isolated from a fibrotic mouse model showed significantly increased miR-let-7c levels compared with control and that overexpression of this miRNA regulated macrophage polarization (76). Expression of miR-124 and miR-223 in macrophages has also been reported to contribute to macrophage polarization (77, 78).

Located on the surface of sentinel cells, such as macrophages, TLRs play a critical role in the innate immune system by recognizing pathogen-associated molecular patterns expressed on pathogens and signaling for the production of cytokine to elicit an immune response. These TLRs participate in macrophage activation and have been shown to induce miR-155, miR-146, miR-147, miR-9, and miR-21 (79, 80). An upregulation of miR-21 has been observed in both primary human airway epithelial cells (41) and in an IL-13 transgenic mouse model with the latter study identifying that the observed miR-21 upregulation was through an IL-13Rα1-dependent mechanism (81). This increase in miR-21 was also associated with inhibited Th1 cytokine signaling (41). Using an ovalbumin-induced miR-21 deficient mouse model, Th1 cytokines were found to be increased (82), supporting the contribution of miR-21 in Th2 type immune responses. One study confirmed that miR-21 expression inhibited murine pulmonary inflammation by suppressing TLR2 signaling (81). When secreted from tumor cells, miR-21 and miR-29a have also been reported to interact with TLRs, specifically TLR7 and TLR8, respectively (83). Upon lipopolysaccharide (LPS) stimulation, miR-146a/b was shown to be induced and predicted to negatively regulate TLR and cytokine signaling (72).

### Influence of miRNA on T-Helper Cell Responses

Macrophage surface activation is induced in the presence of overexpressed miR-125b (84) and in an eosinophilic rhinosinusitis animal model, miR-125b is increased resulting in increased interferon gamma and a Th1 type immune response (85). miR-19a has also been shown to be critical in regulating Th1 type responses through the production of interferon gamma following antigen stimulation in a mouse model (69). Upregulation of miR-19a also caused increased inflammation and promoted a Th2 type response (55). miR-19a is a member of the miR-17–92 cluster, which has been reported to be upregulated during T-cell activation (69, 86). Th17 cell differentiation has also been shown to be regulated by the miR-106-363 cluster (87) and in an experimental autoimmune encephalomyelitis model, Th17 cell-mediated inflammation was shown to be induced by both miR-326 and miR-21 (88, 89).

**Figure 1:**
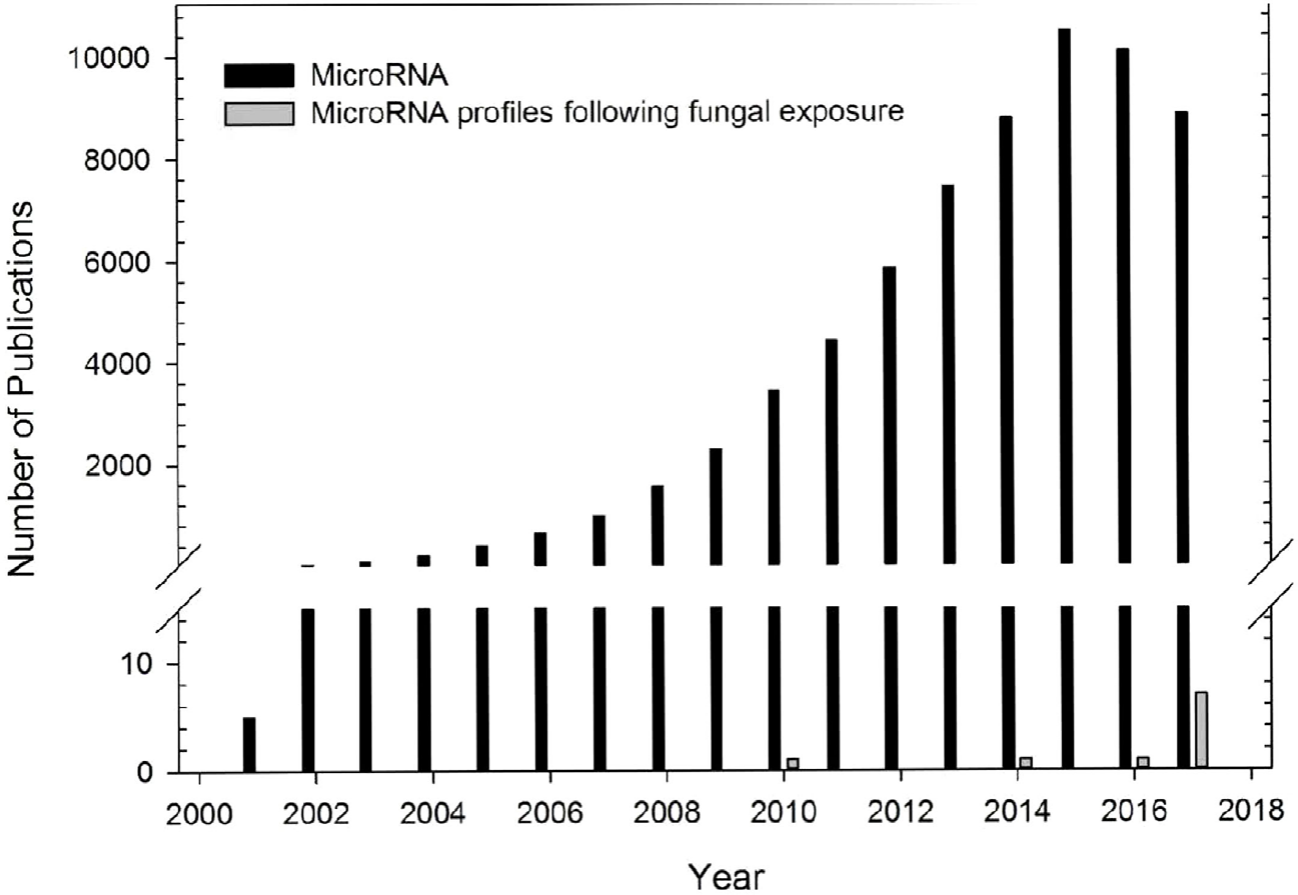
Number of microRNA (miRNA) publications. Number of publications examining miRNA profiles from different diseased models (black bars) or following fungal exposure (gray bars). Data obtained from PubMed search using term “microRNA” for publications using different diseased models. One study in 1972 was not included. Number of publications using a fungal exposed model were obtained from studies included in this review.

In summary, multiple studies have characterized the role of miRNAs on immune processes in a variety of diverse animal models of inflammation, but few studies have evaluated the miRNA profiles following fungal exposures (Figure 1). Investigation of these miRNA profiles could provide insight into the immune mechanisms and regulatory pathways involved in the host response to fungal exposure.

## Fungal Exposure: Role of miRNAs

Several research studies have focused on the miRNA profiles following acute or chronic fungal exposures (Figure 1). Table 1 shows 10 studies that have preliminarily characterized differentially expressed miRNAs following exposure to five clinically relevant fungal species including *A. fumigatus* (22, 90, 91), *C. albicans* (22, 92–94), *C. neoformans* (95), *P. brasiliensis* (96, 97), and *S. chartarum* (98). The paucity of research investigating the regulation of miRNAs on pulmonary and systemic responses to fungal exposure highlights the need for research examining the role miRNAs play in the immunological mechanisms associated with endemic, opportunistic, and environmental fungal exposures.

**Table 1:** Differentially expressed microRNAs following fungal exposure.

miRNA	Regulation	Fungal species	Exposure model	Function in immune response^a^	Reference
let-7 family	↑	*Paracoccidioides brasiliensis*	Inv	Regulator of TLR mediated signaling; Involved in IL-13 production	(97, 99)
miR-125a	↑	*Candida albicans*	Cc	Regulator of macrophage polarization; Enhances classical activation of macrophages	(92, 100)
miR-125b	↑	*P. brasiliensis*	Hu		(96, 101)
miR-126a	↑	*P. brasiliensis*	Inv	Promoter of Th2 immune response	(49, 97)
miR-129	↑	*C. albicans* with *Aspergillus fumigatus*	Cc	Regulation of cell cycle	(22, 102)
miR-130a	↑	*P. brasiliensis*	Inv	Involved in CD8^+^T cell activation	(97, 103)
miR-132	↑	*P. brasiliensis*	Hu	Regulator of TLR2 mediated signaling; induces alternative activation of macrophages	(96, 104)
	↑	*A. fumigatus*	Cc		(90, 101)
	↑	*C. albicans* with *A. fumigatus*	Cc		(22)
miR-146a	↑	*C. albicans*	Cc	Negative regulator of TLR mediated signaling; Induces alternative activation of macrophages	(92, 94)
	↑	*Cryptococcus neoformans*	Cc		(95, 101, 105)
miR-155	↑	*C. albicans*	Cc	Regulator of TLR mediated signaling; Enhances classical activation of macrophages; Promoter of Th2 immune response	(92, 93, 100, 101, 105)
miR-186	↑	*P. brasiliensis*	Hu	Involved in TLR2 mediated signaling	(96, 106)
miR-19b	↑	*P. brasiliensis*	Inv	Promoter of Th17 immune response; T cell proliferation	(97, 107)
miR-20a	↑	*P. brasiliensis*	Inv	Negatively regulates monocyte differentiation	(97, 108)
miR-210	↑	*C. albicans*	Cc	Involved in Th1 and Th17 cell differentiation	(94, 109)
miR-21a	↑	*Stachybotrys chartarum*	Inh	Regulator of TLR mediated signaling; Involved in monocyte, dendritic, macrophage, and Th2 cell differentiation; Involved in macrophage activation	(98, 109, 110)
miR-212	↑	*C. albicans* with *A. fumigatus*	Cc	Involved in B-cell development and Th17 cell differentiation	(22, 111)
miR-23	↓	*A. fumigatus*	Inh	Involved in cell differentiation of B cells; Mediate macrophage polarization	(91, 109)
miR-26b	↑	*P. brasiliensis*	Inv	Increases regulatory T cells	(97, 107)
miR-29a	↓	*A. fumigatus*	Inh	Regulator of natural killer cell function; Involved in C-leptin signaling; Inhibition of Th1 immune response	(91, 112)
miR-29b	↑	*P. brasiliensis*	Hu		(96, 100)
miR-30	↑	*C. albicans*	Cc	Involved in natural killer cell function and B cell activation; Involved in cell function of cytotoxic T cells	(94, 109)
miR-30b	↑	*P. brasiliensis*	Inv, Hu		(96, 97)
miR-301a	↑	*P. brasiliensis*	Inv	Promoter of Th17 immune response; Regulator of TLR mediated signaling	(97, 100, 113)
miR-340	↑	*P. brasiliensis*	Inv	Unknown	(97)
miR-369	↑	*P. brasiliensis*	Inv	Activation of protein translation	(26, 97)
miR-376c	↑	*P. brasiliensis*	Hu	Unknown	(96)
miR-423	↓	*P. brasiliensis*	Hu	Regulator of chemokine expression	(96, 114)
miR-455	↑	*C. albicans*	Cc	Involved in nuclear factor-kappaB signaling	(92, 115)
miR-466k	↑	*P. brasiliensis*	Inv	Unknown	(97)
miR-604	↑	*P. brasiliensis*	Hu	Unknown	(96)
miR-706	↑	*S. chartarum*	Inh	Promotes granulocyte production	(98, 116)

*^a^Immune responses are compiled from different studies utilizing a variety of diseased models and not necessarily from fungal exposure studies. Exposure model abbreviations are Inv, Intravenous administration or through; Inh, Inhalation using a mouse model; Cc, cell culture; Hu, Human patients*.

### miRNA Profiles following *P. brasiliensis* Infection

Paracoccidioidomycosis, caused by the dimorphic fungus *P. brasiliensis*, is a public health burden in Latin America (117). This fungus can be isolated in the form of yeast from infected individuals and armadillos, and has also been sporadically isolated from soil, dog food, and bat feces (118, 119). The disease begins with the inhalation of spores into the lungs that germinate into yeast and cause a primary lung infection or disseminate systemically resulting in oral and cutaneous lesions. To date, two studies have evaluated differentially expressed miRNAs following *P. brasiliensis* exposure in a murine model and in a human model. Turini Gonzales Marioto et al. (97) evaluated the miRNA profiles in mice intravenously administered *P. brasiliensis* and showed that the most upregulated miRNAs at 28 days included miR-126a-5p, miR-340-5p, miR-30b-5p, miR-19b-3p, miR-221-3p, miR-20a-5p, miR-130a-3p, and miR-301a-3p, whereas after 56 days, miRNAs from the let-7 family, as well as miR-26b-5p, and miR-369-3p were the greatest upregulated miRNAs (97). The only miRNA that was upregulated at both time points was miR-466k (Table 1). This study identified differentially expressed miRNAs that are known to contribute to the immune response through T cell function and proliferation, as well as monocyte and erythrocyte differentiation. The contribution of miR-466k on the immune response is unknown; however, this miRNA has been identified in prostate cancer and graft rejection studies (120, 121). Another study examined the miRNA profile in the serum of human patients infected with *P. brasiliensis* and found that of the 752 miRNAs analyzed, 8 were differentially expressed (96). The upregulated miRNAs included miR-132-3p, miR-604, miR-186-5p, miR-29b-3p, miR-125b-5p, miR-376c-3p, and miR-30b-5p, where the only downregulated miRNA was miR-423-3p (Table 1). These miRNAs are known to mediate macrophage polarization or are involved in TLR2 signaling, indicative of a Th1 immune response. Interestingly, both studies reported an upregulation in miR-30b-5p, suggesting a possible biomarker for *P. brasiliensis* infection.

### miR-132 Is Induced by *A. fumigatus* Exposure

*Aspergillus fumigatus* is a commonly encountered pathogenic fungal species and is often found in the soil, occupational environments [i.e., biowaste containment facilities (122, 123)] or indoor environments [i.e., hospitals (124, 125)]. Inhalation of *A. fumigatus* unicellular spores can result in varying degrees of infection, known as aspergillosis, depending on the preexisting conditions of the host. miR-132 has been shown to be induced in human monocytes and dendritic cells following stimulation with *A. fumigatus* compared with control, LPS (90). These datasets suggest a Th2-mediated response, which is further supported by other recent animal models of inhalation exposure to *A. fumigatus* (91).

### Upregulation of miR-146 in *Candida* and *C. neoformans*

Candidiasis, an infection caused by several endogenous *Candida* species, results in varying symptoms depending on the site of infection (126, 127). Candidiasis is among the most common opportunistic fungal infections localized in the gastrointestinal tract (thrush), occluded regions of the hands, feet, and groin, or can develop into invasive candidiasis and disseminate systemically in the blood (candidemia), heart, brain, eyes, and bones. Invasive candidiasis is the most common type of fungal infection in critically ill patients, with approximately 46,000 healthcare-associated cases occurring each year in the United States (126–128). Although *Candida* infections are typically resolved by antifungal therapy, some *Candida* species are resistant or are becoming resistant, such as *C. auris* (129). In murine macrophages stimulated by 10^6^ cells/mL heat killed *C. albicans*, miR-146a and miR-155, as well as miR-455 and miR-125a were upregulated (92), indicative of the involvement of these miRNAs in macrophage polarization.

Similar to the findings of Monk et al. (92), miR-146a was also shown to be upregulated in human monocytic THP-1 cells exposed to *C. neoformans*, inhibiting nuclear factor-κB activation and the release of inflammatory cytokines (95). *C. neoformans* is one of two pathogenic *Cryptococcus* species, and along with *C. gatti*, typically live in the soil surrounding trees and are capable of causing infection following inhalation. Exposure to *Cryptococcus* species usually causes adverse respiratory health effects; however, it can affect other parts of the body such as the brain, known as cryptococcal meningitis (130, 131).

### miRNA Profiles following a Mixed Fungal Exposure

In a model of mixed fungal exposure, Dix et al. co-cultured human monocyte-derived dendritic cells with *A. fumigatus, C. albicans*, and LPS and showed that differentially expressed miRNAs were increased after 6 and 12 h, with a stronger regulation observed after 12 h (22). Twenty six miRNAs were identified to be differently expressed in response to the exposure. The authors also showed that strongly modified miRNAs after exposure to fungi clustered separately from the strongly modified miRNAs exposed to LPS. This clustering pattern suggests that examination of miRNA profiles could distinguish between fungal and bacterial exposure. For example, miR-132 and miR-212-5p were specific to fungal exposure at 6 h time point, whereas miR-132, miR-212, and miR-129-5p were specific to fungal exposure at 12 h time point.

### C-Leptin Receptors and Associated miRNAs

Critical to antifungal innate immunity, Dectin-1 is a surface receptor that recognizes (1,3)-β-D-glucan found on the cell wall of germinating conidia (91, 132). To date, several studies have attempted to explore the regulatory mechanisms involving miRNAs that underlie Dectin-1 associated immune responses. In a murine model of subchronic *A. fumigatus* inhalation exposure, Croston et al. showed that significantly downregulated miR-29a-3p was predicted to regulate C-type lectin domain family 7 member A, the gene that codes for Dectin-1 (91). A recent study found that following exposure to *C. albicans*, Dectin-1 is required for the upregulation of miR-155 in murine macrophages (93). Along with an increase in miR-30-5p and miR-210-3p in THP-1 cells treated with β-glucan isolated from *C. albicans*, Du et al. found that miR-146a was increased upon Dectin-1 stimulation and negatively regulated the resultant inflammatory response (94). Results from the Croston et al. study using a murine model of subchronic *A. fumigatus* inhalation exposure also determined that significantly downregulated miR-23b-3p and miR-145a-5p was predicted to regulate the mannose receptor gene, *Mrc1* (data not reported).

### miRNA Profiles following Exposure to Occupationally Relevant Fungal Species

In order to elucidate the influence of germination on the ensuing immune response, Croston et al. utilized an acoustical generator system to deliver dry fungal spores to mice housed in a multi-animal nose-only inhalation chamber (91). This murine inhalation model reproduces exposures that could be encountered in contaminated indoor or occupational environments (133). Furthermore, this study included a heat inactivated conidia group that was used as a biological control to examine the influence of germination on the miRNA profiles. Along with a downregulation of miR-29a-3p, miR-23b-3p, a miRNA predicted to target *SMAD2*, as well as genes involved in IL-13 and IL-33 responses, was also downregulated following subchronic exposure to a dry aerosol containing viable *A. fumigatus* conidia (91). Furthermore, out of 415 miRNAs detected, approximately 50% were altered in mice exposed to viable versus heat inactivated conidia 48 h post fungal exposure. Taken together, these results demonstrate that *A. fumigatus* germination is an important variable that can lead to the induction of allergic inflammation in the lungs, potentially through an IL-13/IL-33-driven mechanism.

**Figure 2:**
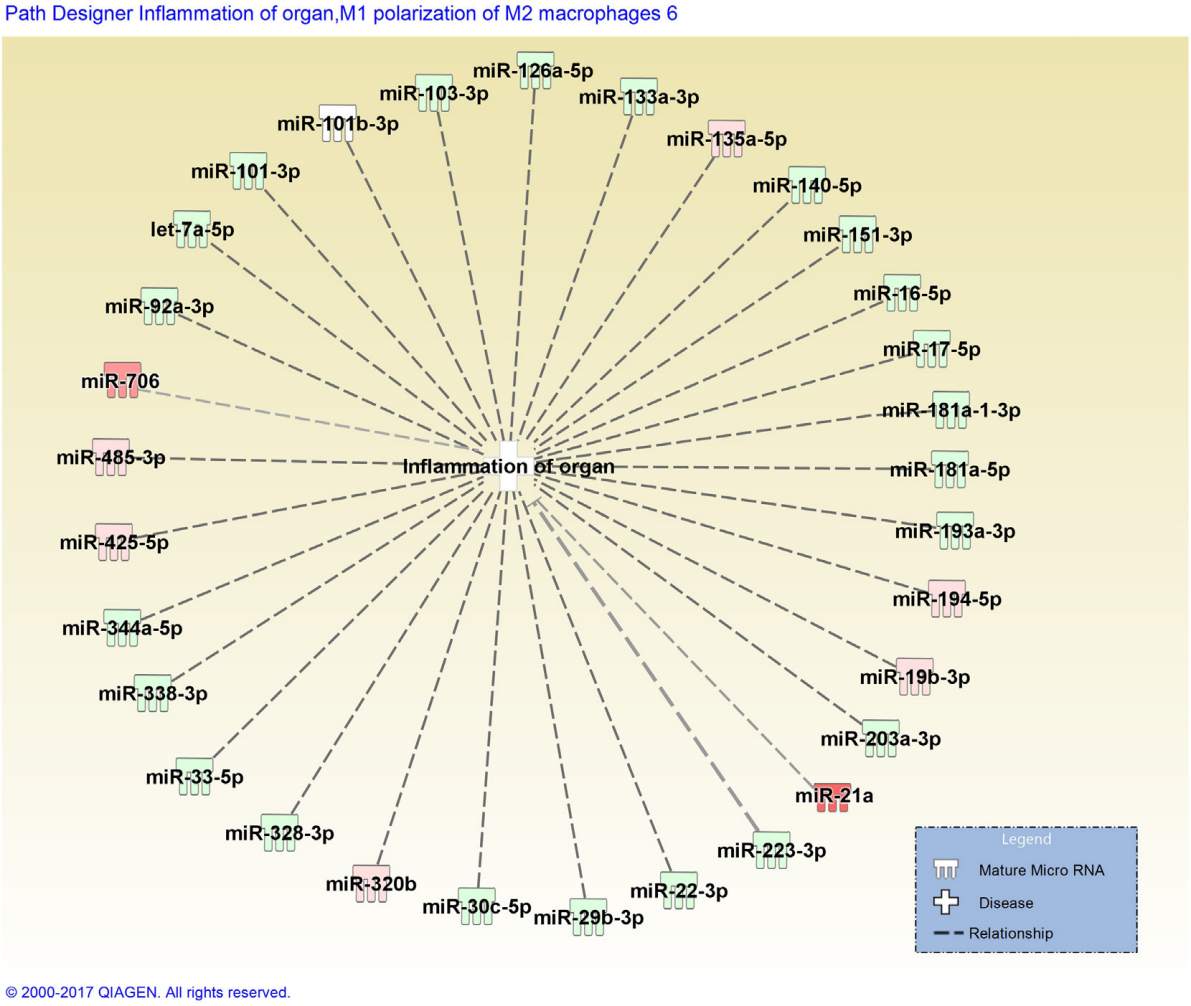
Disease network map generated by Ingenuity Pathway Analysis depicting miRNAs involved in the inflammation of organs. miRNAs are color-coded (red or green for up- and downregulation, respectively) for the expression of miRNAs in *Stachybotrys chartarum* exposed versus control at 13 weeks, 48 h post-exposure. Gray dotted lines represent predicted regulated relationship.

*Stachybotrys chartarum* is a hydrophilic fungal species prevalent in water infiltrated occupational and residential environments. Exposure to *S. chartarum* is currently of heightened public health interest following recent natural disasters, such as floods and hurricanes, that can lead to the contamination of indoor building materials. Following the consensus reports published by the IOM and WHO, the immunological mechanisms that contribute to the host response to fungal contaminant exposure require further elucidation. Recent studies have attempted to characterize critical interactions that influence these pulmonary immunological responses. Croston et al. found that miR-21a was the only miRNA upregulated in murine whole lung homogenate 48 h following subchronic exposure to *S. chartarum* (98) (Figure 2). Although miR-21a is known to promote a Th2 phenotype, a more dominant Th1 phenotype was evident. Since then, miR-706 was also discovered to be upregulated at the same time point. Interestingly, out of 468 miRNA evaluated, only 2 were upregulated with no downregulated miRNAs. These preliminary results suggest that miRNA regulation mechanisms induced by *S. chartarum* vary from *A. fumigatus* in these studies using the same exposure system and the pulmonary immunological responses to this species require further evaluation.

Figure 2 depicts a disease pathway generated by Ingenuity Pathway Analysis (IPA) that includes predicted miRNAs involved in the inflammation of organs. Once miRNA data are uploaded into IPA, the integrated knowledge base predicts associations between miRNAs from the dataset and different disease pathways or biological functions. These predictions are primarily based on previously publish datasets derived from a broad diversity of animal models. The miRNA dataset included in Figure 2 was obtained from murine whole lung homogenate 48 h following subchronic exposure to *S. chartarum* (98). When analyzing the top diseases and biological functions of the miRNAs included in the dataset, a handful were predicted to be involved in an inflammatory response, specifically in organ inflammation, illustrated in Figure 2. The miRNAs are color-coded depending on the respective expression level (red or green for up- and downregulation, respectively). The absence of confirmed associations between miRNAs, evidenced by gray dotted lines, supports the lack of miRNA profile studies following fungal exposure.

To date, only a handful of studies have examined the altered miRNA profiles following fungal exposure; therefore, more researches are required to fully understand the mechanistic influence miRNAs have on the immune response. With the increased interest in studying miRNAs, methodological approaches are becoming more advanced by using next-generation sequencing methods that examine miRNA profiles in more depth and at a higher precision compared with miRNA arrays. Once the more influential miRNAs are identified, strategies can be developed in order to manipulate the host response. With the use of transgenic or knockout animal models, the functionality of miRNAs or genes can be elucidated; however, the manipulation of the genome may in fact alter normal miRNA production or function, contributing to the phenotype of the disease (134). Targeting miRNAs that are upregulated or replacing the expression of miRNAs that are downregulated are potential strategies that could be tested in animal models as a new therapeutic strategy to treat fungal infections and diseases. This targeting strategy could be completed through the use of an anti-miR or a miRNA mimic (135), and may allow for the manipulation of a group of genes or proteins that participate in the progression of the infection or disease. Ultimately, these targeting strategies will help bridge the knowledge gap between the identification of miRNAs and the host responses to fungal exposure, potentially leading to advanced therapeutics to combat adverse effects resulting from exposure to pathogenic fungi.

## Conclusion

In this review, the identification and influence of miRNAs on the host immune responses following fungal exposure were examined. Compared with existing datasets examining miRNA profiles in allergic and inflammatory models, some common differentially expressed miRNAs were identified in fungal exposed models. Influential miRNAs altered in different disease models, such as miR-132, functions to maintain a normal hematopoietic output during an immune response and regulates genes at the beginning of an immune response to regain homeostasis of the immune system. Other common miRNAs identified in multiple inflammatory disease models, including miR-155 and miR-146a, regulates critical genes involved in the host defense system through opposing mechanisms. For example, miR-146a is known to decrease cytokine production and inhibits Th1 cells following an inflammatory stimulus, as well as induces alternative activation of macrophages, whereas miR-155 stimulates both Th1 and Th17 immune responses and induces classical activation of macrophages. Taken together, these miRNAs act in concert to defend the host from infection. In addition to common miRNAs identified in multiple diseased models, the miRNAs that were observed to be differentially expressed specifically in fungal exposed models could potentially serve as biomarkers for fungal exposures.

Recent discoveries in miRNA biology have heightened the research community’s interest in examining the altered genetic profiles in different disease models; however, only a few studies have examined miRNA profiles following fungal exposure. As such, the description of the immune responses to the corresponding miRNAs listed in Table 1 was not all compiled from fungal exposure studies due to the lack of research examining the influence of miRNAs on the immune responses following fungal exposure. Although advancements made in this field have helped elucidate mechanisms underlying host responses to a variety of infections and diseases, further examination of miRNA profiles, specifically in fungal exposed models, is required in order to provide greater mechanistic insight into the immunological response to clinically and environmentally relevant fungal species.

## Author Contributions

TC and BG designed the manuscript; TC drafted the manuscript and prepared figures/tables; TC, AL, DB, and BG revised and edited the manuscript; TC, AL, DB, and BG approved the final version of the manuscript and agree to be accountable for the content of the work.

## Conflict of Interest Statement

The authors declare that the research was conducted in the absence of any commercial or financial relationships that could be construed as a potential conflict of interest.

## Abbreviations

Clec7a, C-type lectin domain family 7 member A; IL, interleukin; LPS, lipopolysaccharide; RNA, ribose nucleic acid; RNAi, RNA interference; mRNA, messenger RNA; miRNA, microRNA; TLRs, Toll-like receptors.
